# Evaluation of Synergistic Antibacterial and Antioxidant Efficacy of Essential Oils of Spices and Herbs in Combination

**DOI:** 10.1371/journal.pone.0131321

**Published:** 2015-07-01

**Authors:** Anwesa Bag, Rabi Ranjan Chattopadhyay

**Affiliations:** Agricultural and Ecological Research Unit, Indian Statistical Institute, Kolkata, India; Second University of Naples, ITALY

## Abstract

The present study was carried out to evaluate the possible synergistic interactions on antibacterial and antioxidant efficacy of essential oils of some selected spices and herbs [bay leaf, black pepper, coriander (seed and leaf), cumin, garlic, ginger, mustard, onion and turmeric] in combination. Antibacterial combination effect was evaluated against six important food-borne bacteria (*Bacillus cereus*, *Listeria monocytogenes*, *Micrococcus luteus*, *Staphylococcus aureus*, *Escherichia coli and Salmonella typhimurium*) using microbroth dilution, checkerboard titration and time-kill methods. Antioxidant combination effect was assessed by DPPH free radical scavenging method. Total phenolic content was measured by Folin-Ciocalteu method. Bioactivity –guided fractionation of active essential oils for isolation of bioactive compounds was done using TLC-bioautography assay and chemical characterization (qualitative and quantitative) of bioactive compounds was performed using DART-MS and HPLC analyses. Cytotoxic potential was evaluated by brine shrimp lethality assay as well as MTT assay using human normal colon cell line. Results showed that among the possible combinations tested only coriander/cumin seed oil combination showed synergistic interactions both in antibacterial (FICI : 0.25-0.50) and antioxidant (CI : 0.79) activities. A high positive correlation between total phenolic content and antibacterial activity against most of the studied bacteria (R^2^ = 0.688 – 0.917) as well as antioxidant capacity (R^2^ = 0.828) was also observed. TLC-bioautography-guided screening and subsequent combination studies revealed that two compounds corresponding to R_f_ values 0.35 from coriander seed oil and 0.53 from cumin seed oil exhibited both synergistic antibacterial and antioxidant activities. The bioactive compound corresponding to R_f_ 0.35 from coriander seed oil was identified as linalool (68.69%) and the bioactive compound corresponding to R_f_ 0.53 from cumin seed oil was identified as p-coumaric acid (7.14%) by DART-MS and HPLC analyses. The coriander/cumin seed oil combination did not show any cytotoxic effect both in brine shrimp lethality as well as human normal colon cell line assays. The LC_50_ in brine shrimp lethality assay was found to be 4945.30 μg/ml and IC_50_ in human normal colon cell line was > 1000 μg/ml. The results provide evidence that coriander/cumin seed oil combination might indeed be used as a potential source of safe and effective natural antimicrobial and antioxidant agents in pharmaceutical and food industries.

## Introduction

Food-borne disease is an increasingly major public health problem all over the world. Microbial contamination and food oxidation are the two most important factors for developing food-borne diseases and food spoilage [1]. Prevention of microbial contamination and food oxidation is usually achieved by synthetic food preservatives. These synthetic food preservatives can be categorised mainly into two general types, antimicrobials that inhibit the growth of microbes, and antioxidants that slow the air oxidation of fats and oils in foods which lead to rancidity [2]. But these synthetic food preservatives are harmful to human health and have many side effects including headache, nausea, weakness, mental retardation, seizures, cancer and anorexia along with growing concern of emergence of multidrug resistant microbes [3]. Therefore, there is growing interest in the development of safe and effective natural food preservatives.

Spices and herbs have been used traditionally for thousands of years by many cultures not only as flavouring agents but also as food preservatives. They are generally recognised as safe (GRAS) because of their traditional use without any documented detrimental impact. They are also inexpensive, show better patient tolerance and are readily available for low socioeconomic population [4, 5]. In view of their beneficial effects, spices and herbs are gaining importance in recent years as potential source of natural food preservatives. But the main obstacle for using spices and herbs as food preservatives is that their high concentration is required in food to inhibit the microbial growth as well as oxidation. This high concentration of spices and herbs causes negative organoleptic effects i.e. alter the taste, colour, odour and texture of foods and limit their use in food preservation system [6]. This negative organoleptic effect of spices and herbs should be addressed in order to facilitate their use in food preservation system as well as to develop safe and potent natural antimicrobial and antioxidant food preservatives from them. The individual effects of essential oils of spices and herbs on antimicrobial activity against food-borne bacteria as well as their antioxidant activity have been extensively reported by several workers [7–9]. But their combination effects on both antimicrobial and antioxidant activities seem to be dubious. This approach may increase their antimicrobial and antioxidant efficacy at sufficiently low concentration by taking their advantages of possible synergistic interactions. This synergistic interaction may reduce their adverse side effects as well as negative organoleptic effects in food and facilitate their use in food preservation system. The present investigation has been designed to shed some light on these important aspects.

## Materials and Methods

### Collection, identification and processing of plant materials

Ten commonly used spices and herbs [bay leaf (BL/01/14), black pepper (seed) (BP/02/14), coriander (seed) (CS/03/14), coriander (leaf) (CL/04/14), cumin (seed) (CU/05/14), garlic (bulb) (GA/06/14), ginger (rhizome) (GI/07/14), mustard (seed) (MS/08/14), onion (bulb) (ON/09/14) and turmeric (rhizome) (TU//10/14)] were purchased from the local market (Burrabazar) in Kolkata, West Bengal, India). These spices and herbs were authenticated by a botanist and their voucher specimens were deposited at Agricultural and Ecological Research Unit, Indian Statistical Institute, Kolkata, India. The spices and herbs were washed thoroughly in tap water, dried and milled to powder for extraction of essential oils.

### Extraction of essential oils by hydrodistillation

The essential oils of selected spices and herbs were obtained by hydrodistillation using a Clevenger type apparatus. Briefly, 100 g powder of each of the spices and herbs was subjected to hydrodistillation for 3h. The obtained essential oils were dried over anhydrous sodium sulphate and stored in the dark at 4°C until used. For experimental purposes, the essential oils were reconstituted in 0.5% dimethylsulfoxide (DMSO) with Tween 80 (0.02% v/v for easy diffusion). Yields of essential oils are shown in Table 1.

**Table 1:** Spices and herbs used for the study and yield of their essential oils.

Scientific Name	Common Name	Family	Parts used	Yields of essential oils (%)
*Allium cepa*	Onion	Allium	bulb	0.19
*Allium sativum*	Garlic	Allium	bulb	0.30
*Brassica nigra*	Mustard	Brassicaceae	seed	0.54
*Coriandrum sativum*	Coriander	Apiaceae	leaf	0.42
*Coriandrum sativum*	Coriander	Apiaceae	seed	0.73
*Cuminum cyminum*	Cumin	Apiaceae	seed	0.67
*Curcuma longa*	Turmeric	Zingiberceae	rhizome	0.37
*Laurus nobilis*	Bay leaf	Lauraceae	leaf	0.20
*Piper nigrum*	Black pepper	Piperaceae	seed	0.39
*Zingiber officinale*	Ginger	Zingiberaceae	rhizome	0.40

### Microorganisms

Forty one food-borne bacterial strains isolated from food samples following bacteriological, morphological and biochemical characterization [10] were used in this study. The strains included *Bacillus cereus* (6 strains), *Listeria monocytogenes* (8 strains), *Micrococcus luteus* (7 strains), *Staphylococcus aureus* (7 strains), *Escherichia coli* (7 strains) and *Salmonella typhimurium* (6 strains). Two reference standard bacterial strains used were *Bacillus cereus* (MTCC 1272) and *Salmonella typhimurium* (MTCC 3224). These reference standard strains were procured from Institute of Microbial Technology, Chandigarh, India. All the strains were maintained on selective agar slants following standard guidelines [11].

### Inoculum preparation

The inoculum size of the test bacterial strains was standardized according to the National Committee for Clinical Laboratory Standards guidelines [11]. The bacterial strains used in this study were inoculated in selective broths media (HiMedia, Mumbai, India) and incubated at respective temperature (30°C / 37°C) following standard guidelines and kept in a shaker water bath for 3–6 h until the culture attained a turbidity of 0.5 McFarland Unit. The final inoculum size was adjusted to 5 × 10^5^ CFU/ ml.

### Antibacterial susceptibility screening

#### Determination of inhibition zone diameter (IZD)

Susceptibility test was performed by a modified agar well diffusion method [12]. Briefly, one ml of inoculum (5 × 10^5^ CFU/ml) was spread evenly with a glass rod spreader on selective nutrient agar (HiMedia, Mumbai, India) plates and six mm diameter wells were bored on the surface of agar plates. 100 μl of 10 mg/ml reconstituted each essential oil was pipetted into wells. After holding the plates at room temperature for 2h to allow diffusion of essential oils into the agar, they were incubated at respective temperature (30°C / 37°C) for 24h. Inhibition zone diameter (IZD) was measured to the nearest millimetre (mm). Amikacin (30μg) (HiMedia, Mumbai, India) was used as experimental positive control and 0.5% DMSO as negative control. The tests were performed in triplicate for each microorganism used. Only essential oils that showed promising antibacterial activity (IZD ≥ 11 mm) [13] against at least one of the studied bacteria were considered as active essential oils and selected for antibacterial and antioxidant combination studies.

### Antibacterial combination study

#### Determination of minimum inhibitory concentration (MIC)

For antibacterial combination study, at first MICs of active essential oils alone against the studied bacteria were determined in flat-bottom 96-well micro-titre plates containing selective broth media (90 μl) in each well. The essential oils were diluted two-fold serially (1000 μg/ml to 15.6 μg/ml) with selective broth from which 100 μl solution was given in each well containing 90 μl broth. 10 μl of working inoculum suspension (5×10^5^ CFU/ml) was added to the wells. A number of wells were reserved in each plate for control of sterility (no inoculum added), inoculum viability (no sample solution added) and DMSO inhibitory effect. The plates were then incubated for 24 h at respective temperature (30°C / 37°C). After incubation, 40 μl of 0.4 mg/ml p-iodonitrotetrazolium violet (Sigma-Aldrich) solution (INT) was added in each well and further incubated for 6h. The micro-titre plates with bacteria were then examined to determine a colour change. Viable microorganisms interact with the INT solution to cause a colour change from faint yellow to red-purple colour. The lowest dilution with no colour change was considered as the MIC for that individual oil [14]. The tests were performed in triplicate.

#### Determination of Fractional Inhibitory Concentration Index (FICI)

Fractional inhibitory concentration index was determined by checkerboard titration method. For this, after determining the individual MICs of active essential oils, their MICs in combination were determined in microbroth dilution method [14]. Briefly, selective broth media (90 μl) and 10 μl of working inoculum (5 × 10^5^ CFU/ml) were added in each well of micro-titre plates. 100 μl of test essential oils in combination (1:1 v/v) of different concentrations ranging from 1/32 × MIC to 4 × MIC was added to the wells. The growth conditions were the same as previously mentioned to determine the individual MIC. Fractional inhibitory concentration indices (FICI) were calculated using the formula: FICI = (MIC of EO_A_ in combination with EO_B_ / MIC of EO_A_ alone) + (MIC of EO_B_ in combination with EO_A_/ MIC of EO_B_ alone). Where EO_A_ and EO_B_ are tested two different essential oils. The results were interpreted according to FIC_indices_ as follows: FICI ≤ 0.5: Synergy; 0.5 < FICI ≤ 4: Additive; and FICI > 4: Antagonistic [15]. All the experiments were repeated thrice.

#### Time-kill assay

Synergistic activity of essential oils in combination as observed in checkerboard titration method was confirmed by time-kill assay. Here, the combination of ¼ × MIC was applied. Briefly, 10 ml selective broth, 100 μl of essential oils in combination (1:1 v/v) at ¼ × MIC concentration and 10 μl inoculum (5 × 10^5^ CFU/ml) were taken in glass tubes. The tubes were incubated at respective temperature (30°C / 37°C) for 24h. 500 μl sample was removed from culture tubes at 0, 3, 6 and 24h of incubation, diluted serially and 100 μl of diluted samples were inoculated on selective media and incubated at 30°C / 37°C for 24h in order to determine viable cell count. The inoculum seeded broth without essential oil served as control. Viable counts were calculated to give CFU/ml and kill-curves were plotted with time against logarithm of the viable count. Each experiment was repeated thrice. Synergy was defined as > 100-fold or >2log_10_ decrease in colony count at 24 h by the combination when compared with their single agent [15].

### Antioxidant combination study

#### DPPH free radical scavenging assay

Free radical scavenging activity of active essential oils alone and in combination (1:1 v/v) was evaluated quantitatively using 1,1-diphenyl-2-picrylhydrazyl (DPPH) free radical scavenging assay method [16]. Briefly, 100 μl of active essential oils in varying concentrations (25 μg/ml–250 μg/ml) were taken in test tubes and 3.9 ml of 0.1mM solution of DPPH in methanol was added to these tubes and shaken vigorously. The tubes were then allowed to stand in dark at room temperature for 30 min. The control was prepared as above without the essential oil and methanol was used for zero adjustment. Absorbance of the samples were measured at 517 nm. Inhibition of the DPPH radical by the active essential oils alone and in combination was calculated according to the following formula.

$$\left(\text{\%}\right)\text{Free radical scavenging }=\;\frac{\left(\text{Ablank}-\text{Asample}\right)\times 100}{\text{Ablank}}$$

Where A_sample_ is the absorbance of DPPH solution after reacting with a given concentration of essential oil and A_blank_ is the absorbance of DPPH solution with methanol blank instead of essential oil. The percentage of DPPH radical scavenging capacity was plotted against the concentration of essential oils alone and in combination and their IC_50_ values (the concentration required for scavenging 50% of the DPPH) were calculated. All tests were performed in triplicate.

#### Determination of antioxidant combination Index (CI)

To investigate the possible synergistic antioxidant activity between the active essential oils, an isobologram analysis based on the median effect principle (IC_50_) was performed. The classical isobologram-combination index equation (CI) was used for analyzing the data [17].

$$\text{CI }=\frac{\left(D\right)1}{\left(Dx\right)1}+\frac{\left(D\right)2}{\left(Dx\right)2}$$

Where (D)_1_ and (D)_2_ are the doses (IC_50_ values) of two active essential oils in combination; (Dx)_1_ and (Dx)_2_ are the doses (IC_50_ values) of two active essential oils individually. On the basis of CI values, the type of antioxidant interactions were interpreted as follows. CI < 1: synergistic; CI = 1: additive; CI > 1: antagonistic.

### Estimation of total phenolic content

Total phenolic content of active essential oils was estimated by Folin-Ciocalteu method following a slight modification [18]. Gallic acid was used as a reference standard for plotting calibration curve. A volume of 0.5 ml of essential oils from 100 μg/ml was mixed with 1 ml of Folin-Ciocalteu reagent (diluted 1:10 with deionized water) and was shaken thoroughly. After 3 min, 3 ml of Na_2_CO_3_ solution (2%) was added and the mixture was allowed to stand for 2h with intermittent shaking for colour development. The absorbance of the resulting blue colour was measured at 760 nm. The total phenolic content was determined from the linear equation of a standard curve prepared with different concentrations of gallic acid. The content of total phenolic compounds was expressed as mg gallic acid equivalent /g (mg GAE /g) of dry mass.

### TLC bioautography-guided isolation of bioactive compounds

TLC bioautography-guided screening for detection and isolation of antibacterial and antioxidant compounds from essential oils that showed synergistic interactions was carried out using the previously identified bacteria *B*. *cereus* MTCC 1272 (that induced highest effectiveness in the well diffusion assay) following the methods described below.

- **Analytical TLC for retention factor (R**_**f**_ **) determination.** Analytical TLC was carried out on TLC plates (5 × 20 cm, 0.25 mm thickness, silica gel G 60 F_254_, Merck, Darmstadt, Germany). A 5 μl of 10 mg/ml concentration of test essential oil was spotted onto the silica gel plate and allowed to dry for a few minutes. Afterwards the plate was developed with toluene: ethyl acetate (95:5 v/v) solvent mixture in a presaturated glass chamber. The developed plate was then air-dried and the spots were inspected under UV light (254 nm) and also by visualization by spraying with p-anisaldehyde—sulphuric acid reagent followed by heating at 110°C for 5 min. The R_f_ values of separated compounds were determined.
- **TLC Bioautography for bioactivity screening.** Bioautographic evaluation was conducted in order to check the antibacterial and antioxidant activity of separated compounds on TLC plate (5 × 20 cm, 0.25 mm thickness, silica gel G 60 F_254_, Merck, Darmstadt, Germany). A 5 μl of 10 mg/ml concentration of test essential oil was spotted on two plates (one plate was used for antibacterial and another for antioxidant activity screening) and allowed to dry for a few minutes. The plates were developed with toluene: ethyl acetate (95:5 v/v) solvent mixture in a presaturated glass chamber and air-dried. For the screening of antibacterial activity, the developed air-dried plate was placed in a sterile petridish, then 0.1 ml inoculum of *B*. *cereus* (5 × 10^5^ CFU/ml) for every 10 ml of melted nutrient agar (HiMedia, Mumbai, India) was distributed over the plate. After solidification of the medium, the plate was incubated for 24 h at 25°C. Subsequently, bioautograms developed were sprayed with 0.2 mg/ml p-iodonitotetrazolium violet (INT) solution. Inhibition zones were observed as clear spots against a purple background on the TLC plate [19]. For the screening of antioxidant capacity, the developed air-dried plate was sprayed with 2.54 mM DPPH in methanol and were further air-dried for 30 min after spraying. Bands with antioxidant capacity were observed as yellow spots on a purple background on the TLC plate [20].
- **Preparative TLC for isolation of bioactive compounds.** A streak of test essential oil was applied manually on a preparative TLC glass plate (20 × 20cm, 1 mm thickness) (Sigma-Aldrich) and allowed to dry for a few minutes. After air drying the plate was developed using the same solvent mixture as used in the analytical TLC in a presaturated glass chamber. In each experiment, two sets of plates were used in parallel. One of the plates from each set of experiment was sprayed with INT solution (for antimicrobials) and DPPH radical (for antioxidants), as described above and the bands that showed antibacterial or antioxidant activity were scrapped off carefully from the second plate of each set of experiment. Then the active constituents were repeatedly eluted from the scrapped silica gel with dichloromethane. The samples were then centrifuged (12000 × g, 15 min) to remove the silica gel and the supernatants were collected. The supernatants were filtered through 0.22 μm filter and dried *in vacuo*.
- **Antibacterial and antioxidant combination study of isolated bioactive compounds.** A combination study was done to evaluate the possible synergistic antibacterial and antioxidant efficacy of isolated bioactive compounds from coriander and cumin seed oil that exhibit both antibacterial and antioxidant activities individually in TLC-bioautography assay. The combination study of isolated bioactive compounds was performed following the same procedure as mentioned above for crude oil. The compounds that showed synergistic antibacterial and antioxidant activities in combination study were subjected to DART-MS analysis for identification of compounds.
- **Identification of isolated bioactive constituents by DART-MS analysis.** For identification of bioactive compounds, samples were subjected to DART-MS profiling on a JEOL AccuTOF JMS-T100LC Mass Spectrometer having a DART (Direct Analysis in Real Time) ion source. Samples were subjected as such in front of DART source. Dry helium was used with 4 LPM flow rate for ionization at 350°C. The orifice 1 was set at 28 V and spectral data were recorded.
- **Quantitation of identified compounds by HPLC.** For quantitation, the identified compounds from DART-MS were then subjected to HPLC analysis on Shimazdu Prominence HPLC system (Shimadzu Corporation) with two mobile phases (i) acetonitrile: water (55:45 v/v) and (ii) water: methanol: glacial acetic acid (65:34:1 v/v). The chromatograms were obtained for 30 minutes with mobile phases at the flow rate of 1.0 ml/min to get a steady base line. The column temperature was set at 25°C and the detection wavelengths were 210 nm and 310 nm for two identified compounds [21,22]. A C18 reversed-phase column (4.6 x 250 mm, 5 μm particle size) was used. Aliquots of standard solutions (0.2–1.0 ml; 100μg/ml) with mobile phases were transferred to a series of 10 ml capacity volumetric flasks to get the concentration ranging 2–10 μg/ml. 20 μl of each calibration standard was injected into the column. Peak areas of each solution were recorded. Calibration curves were plotted between concentration and peak area response. 20 μl of each sample solution prepared from identified compounds with mobile phase and filtered through 0.22 μm membrane filter were injected individually into the column and the area of each peak was recorded duly maintaining the ambient experimental conditions as followed by standard solutions. The amount of identified bioactive compounds in coriander and cumin seed oils were computed from their calibration curves. The samples were analyzed in triplicate.

### Cytotoxicity screening

The cytotoxic potential of the combination of essential oils that showed synergistic interactions was tested by brine shrimp lethality assay as well as MTT assay using human normal colon line.

- **Brine shrimp lethality assay.** The brine shrimp lethality bioassay was used to evaluate the cytotoxic potential of combined essential oils that showed synergistic interactions following the method of Meyer et al [23]. Briefly, artificial sea water was prepared by dissolving 38 g of sea salt in 1 Lit of distilled water for hatching the shrimp eggs. Brine shrimp eggs (*Artemia salina*) were incubated in artificial sea water in a specially designed two-compartment plastic tray under a 60 W lamp, providing direct light and warmth (24°C–26°C). 48 hours were allowed for the shrimp eggs to hatch and mature as nauplii (larva). This was facilitated by attracting the shrimps from one compartment to another compartment of the tray with a light source. 4.5 ml of artificial sea water was taken in eight test tubes and 10 nauplii were added to each of the tubes. Then 0.5 ml of essential oils in combination (1:1 v/v) of different concentrations (0.1–6.4 mg/ml) was added to seven tubes. The control tube devoid of essential oils. All tubes were incubated for 24 h at room temperature. Number of nauplii alive after 24 h was counted with the help of magnifying glass. The percentage mortality of brine shrimp nauplii was calculated. Using probit analysis, LC_50_ (lethal concentration, 50%) was assessed at 95% confidence intervals.
- MTT assay using human normal colon cell line

#### Cell culture

Human normal colon cell line was obtained from American Type Culture Collection (ATCC, USA) and maintained in EMEM medium which was supplemented with 10% fetal bovine serum (FBS), penicillin (100 units/ml), streptomycin (100 μg/ml) and 1% sodium pyruvate. The cells were incubated at 37°C in a humidified 5% CO_2_ incubator.

#### MTT assay

Cytotoxic potential of essential oil combination that showed synergistic interactions was tested in triplicate by MTT [3-(4,3-dimethylthiazole-2-yl)-2,5-diphenyltetrazolium bromide] assay [24] using human normal colon cell line (CCD-18Co) with slight modification. Briefly, after being harvested from culture flasks the cells (100μl) were seeded at a density of 1 × 10^5^ cells/ml in each well of 96 well plate containing 100 μl of fresh growth medium per well and cells were permitted to adhere for 24h at 37°C. The medium was removed after 24 h of incubation and 100 μl of fresh medium containing different concentrations (15.6 μg/ml-1000 μg/ml) of essential oil combination (1:1) were added. To control wells only culture medium (100μl) was used. Following 72 h of incubation, 20 μl of MTT (5 mg/ml) was added into each well and further incubated for another 4h. The formation of insoluble purple formazan from yellowish MTT by enzymatic reduction was dissolved in DMSO (100 μl) after removal of medium. The plates were shaken for 5 min and the absorbance was measured in a microplate reader at 570 nm with 630 nm as reference wavelength. The percent cell inhibition was determined using the following formula: $\text{\% Cell inhibition}\;=\;100-\frac{\text{Absorbance of treated cells}}{\text{Absorbance of control cells}}\;\times 100$ A dose-response curve was plotted from which IC_50_ was calculated.

### Statistical analysis

Data were statistically analysed using SPSS software: Version 18.0. A one-way analysis of variance (ANOVA) followed by Tukey’s range test was applied for analysis of data with the level of significance set at P < 0.05.

## Results and Discussion

Essential oils are complex mixtures of a wide variety of components and have long been recognized for their antimicrobial and antioxidant properties. They show promising antimicrobial activity against a number of microbes including food-borne pathogens and spoilage bacteria and also exhibited antioxidant activity when administered alone *in vitro* [6]. But their antimicrobial and antioxidant combination effects seem to be scarce. Generally the drug combinations have proven to be an essential feature of antimicrobial and antioxidant treatment due to a number of important considerations viz. (i) they increase activity through the use of compounds with synergistic or additive activity; (ii) they thwart drug resistance; (iii) they decrease required doses, reducing both cost and adverse/toxic side effects and (iv) they increase the spectrum of activity.

From the foregoing findings, it was observed that among the ten tested essential oils only three essential oils (coriander, cumin and mustard seed oils) showed promising antibacterial activity (IZD ≥ 11 mm) against most of the studied bacteria (Table 2). These three active essential oils were then subjected to antimicrobial and antioxidant combination study with a view to elucidate their possible synergistic antibacterial and antioxidant potential, if any. In antibacterial combination study, among the three tested combinations (coriander/cumin, coriander/mustard and cumin/mustard), only coriander/cumin combination showed synergistic interaction (FICI: 0.25–0.50) against the studied bacteria except *S*. *typhimurium* and *M*. *Luteus* where it showed additive effect (FICI: 0.75–0.81). Other tested combinations showed additive effect (FICI: 0.75–2.25) against all the studied bacteria. No antagonistic effect was observed (Tables 3 & 4).

**Table 2:** Inhibition Zone Diameter of essential oils of some selected spices and herbs against food-borne bacteria using agar well diffusion assay.

Essential oils of spices and herbs	IZD (mm)							
	*B*. *cereus*	*L*. *monocytogenes*	*M*. *luteus*	*S*. *aureus*	*E*. *coli*	*S*. *typhimurium*	*B*. *cereus(MTCC 1272)*	*S*. *typhimurium(MTCC 3224)*
Bay leaf	8.17 ± 1.09	8.48 ± 1.08	7.76 ± 2.98	8.29 ± 1.00	7.48±1.28	5.83 ± 0.98	10.33±0.57	5.33 ± 1.15
Black pepper	9.89 ± 1.32	8.28 ± 0.89	5.95 ± 1.02	7.10 ± 1.04	7.90±1.13	6.22 ± 1.00	9.00±1.00	8.66 ± 1.15
Coriander (leaf)	6.94 ± 0.93	6.04 ± 1.09	9.00 ± 1.41	9.33 ± 1.35	5.10±0.88	4.89 ± 1.18	9.66±0.57	8.00 ± 1.00
Coriander (seed)	25.0±1.97*	17.92 ± 1.11*	20.14±1.23*	23.24±1.22*	16.10±1.04*	11.61 ± 1.14*	26.33±0.57*	15.33 ± 0.57*
Cumin	22.33±1.28*	16.16 ± 1.10*	18.10±1.13*	20.86±1.06*	12.95±1.07*	10.78 ± 1.30	25.00±1.00*	13.33 ±0.57*
Garlic	9.00 ± 1.18	6.72 ± 1.10	7.86 ± 1.15	9.00 ± 1.09	6.86±1.15	5.61 ± 0.97	9.33±1.52	8.66 ± 1.15
Ginger	9.11 ± 1.13	9.00 ± 0.95	6.86 ± 1.06	8.90 ± 1.30	8.00±1.09	6.61 ± 1.29	9.33±0.57	7.66 ± 0.57
Mustard	20.0 ± 1.23*	14.28 ± 1.20*	15.95±1.11*	18.95±1.07*	11.90±1.33*	11.67 ± 1.02*	23.33±0.57*	11.33 ± 0.57*
Onion	5.83 ± 1.09	5.20 ± 1.19	5.38 ± 1.20	6.90 ± 1.26	5.81±0.92	4.78 ± 1.11	6.33±1.15	4.66 ± 1.52
Turmeric	10.0 ± 1.02	8.08 ± 1.15	9.05 ± 1.32	9.90 ± 1.13	8.00±1.22	6.94 ± 1.05	10.66±1.15	7.66 ± 1.52
Amikacin (Positive control)	27.0 ± 1.28	21.04 ± 1.39	24.05±1.28	30.14±1.23	18.29±1.05	16.83 ± 1.09	29.33±0.57	20.33 ± 1.52
DMSO (Negative control)	-	-	-	-	-	-	-	-

Results are Mean ± S.D. of triplicate experiments.

n = 6, 8, 7, 7, 7, 6 for *B. cereus*, *L. monocytogenes*, *M. luteus*, *S. aureus*, *E. coli* and *S. typhimurium* respectively.

*Sensitive (IZD ≥11 mm: Bauer et al, 1966)

**Table 3:** Minimum inhibitory concentration values of essential oils of spices against food-borne bacteria using microbroth dilution assay.

Spices and herbs	MIC (mg/ml)					
	*B*. *cereus*	*L*. *monocytogenes*	*M*. *luteus*	*S*. *aureus*	*E*. *coli*	*S*. *typhimurium*
Coriander (seed)	0.05 ± 0.03	0.20 ± 0.12	0.33 ± 0.12	0.16 ± 0.06	0.14 ± 0.10	0.19 ± 0.06
Cumin (seed)	0.11 ± 0.06	0.31 ± 0.10	0.29 ± 0.10	0.13 ± 0.07	0.30 ± 0.10	0.38 ± 0.12
Mustard (seed)	0.15 ± 0.05	0.33 ± 0.17	0.41 ± 0.33	0.10 ± 0.03	0.40 ± 0.14	0.45 ± 0.09

n = 6, 8, 7, 7, 7, 6 for *B. cereus*, *L. monocytogenes*, *M. luteus*, *S. aureus*, *E. coli* and *S. typhimurium* respectively

Results are Mean ± SD of triplicate experiments

**Table 4:** Combination effects of essential oils of spices against food-borne bacteria using checkerboard titration method.

Food-borne bacteria	Combinations								
	Coriander (A) + Cumin (B)			Coriander (A) + Mustard (C)			Cumin (B) + Mustard (C)		
	FIC	FICI	Remarks	FIC	FICI	Remarks	FIC	FICI	Remarks
***B*. *cereus***	0.062 (A)	0.25	S	0.062 (A)	0.81	ADD	0.188 (B)	1.00	ADD
	0.188 (B)			0.750 (C)			0.812 (C)		
***L*. *monocytogenes***	0.125 (A)	0.31	S	0.125 (A)	1.00	ADD	0.625 (B)	1.50	ADD
	0.188 (B)			0.875 (C)			0.875 (C)		
***M*. *luteus***	0.062 (A)	0.81	ADD	0.625 (A)	1.50	ADD	0.375 (B)	1.50	ADD
	0.750 (B)			0.875 (C)			1.125 (C)		
***S*. *aureus***	0.250 (A)	0.50	S	0.250 (A)	0.75	ADD	0.125 (B)	1.25	ADD
	0.250 (B)			0.500 (C)			1.125 (C)		
***E*. *coli***	0.125 (A)	0.50	S	0.125 (A)	1.25	ADD	0.375 (B)	1.50	ADD
	0.375 (B)			1.125 (C)			1.125 (C)		
***S*. *typhimurium***	0.250 (A)	0.75	ADD	0.250 (A)	2.00	ADD	0.500 (B)	2.25	ADD
	0.500 (B)			1.750 (C)			1.750 (C)		

n = 6, 8, 7, 7, 7, 6 for *B. cereus*, *L. monocytogenes*, *M. luteus*, *S. aureus*, *E. coli* and *S*. *typhimurium* respectively. S: Synergistic; ADD: Additive

**Fig 1:**
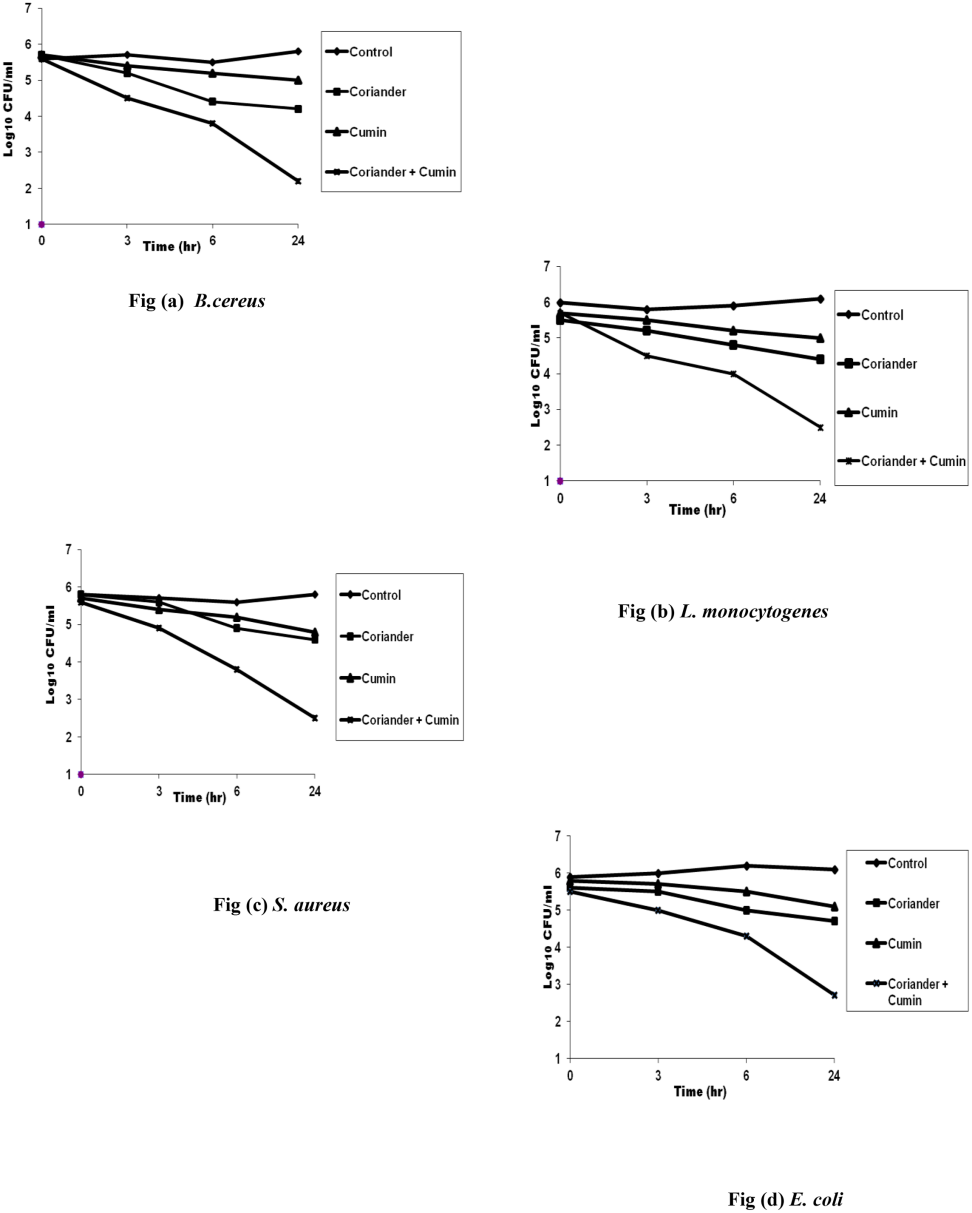
Time-kill curves of coriander/cumin seed oil combination against food-borne bacteria (*B*. *cereus*, *L*. *monocytogenes*, *S*. *aureus* and *E*. *coli*).

In order to confirm the synergistic antibacterial activity of coriander/cumin combination, time-kill assay was performed. Here, coriander/cumin combination reduced the bacterial colony count by >2log_10_ in comparison with the bacterial colony count of their individual effects at 24h (Fig 1). These findings confirmed the synergistic antibacterial activity of coriander/cumin combination of previous experiment.

DPPH assay has been largely used as a quick, reliable and reproducible parameters for screening *in vitro* antioxidant activity of pure compounds as well as plant extracts. The effect of antioxidants on DPPH radical scavenging was thought to be due to their hydrogen donating ability. The reduction capacity of DPPH radical is determined by the decrease in absorbance induced by antioxidants [25]. The test essential oils were able to reduce the stable radical DPPH to the yellow coloured diphenylpicrylhydrazine suggesting their antioxidant activity. On the basis of antioxidant combination index (CI), coriander/cumin seed oil combination showed synergy (CI = 0.79) whereas other combinations showed additive effects (CI = 1.00) (Table 5) which indicates that the proton donating ability of coriander /cumin seed oil combination is high at low concentration over other tested combinations.

**Table 5:** Antioxidant combination effects of active essential oils of spices.

Treatment	IC_50_ (μg/ml)	CI_1_ = (D)_1_ / (Dx)_1_	CI_2_ = (D)_2_ / (Dx)_2_	CI = CI_1_ + CI_2_	Remarks
Coriander	150.62	-	-	-	-
Cumin	163.50	-	-	-	-
Mustard	155.16	-	-	-	-
Coriander + Cumin	62.52	0.41	0.38	0.79	Synergistic
Coriander + Mustard	76.52	0.51	0.49	1.00	Additive
Cumin + Mustard	80.42	0.49	0.51	1.00	Additive

(D)_1_ and (D)_2_ are the doses of two active essential oils in combination; (Dx)_1_ and (Dx)_2_ are the doses two active essential oils individually.

CI <1: synergistic; CI = 1: additive; CI > 1: antagonistic

**Fig 2:**
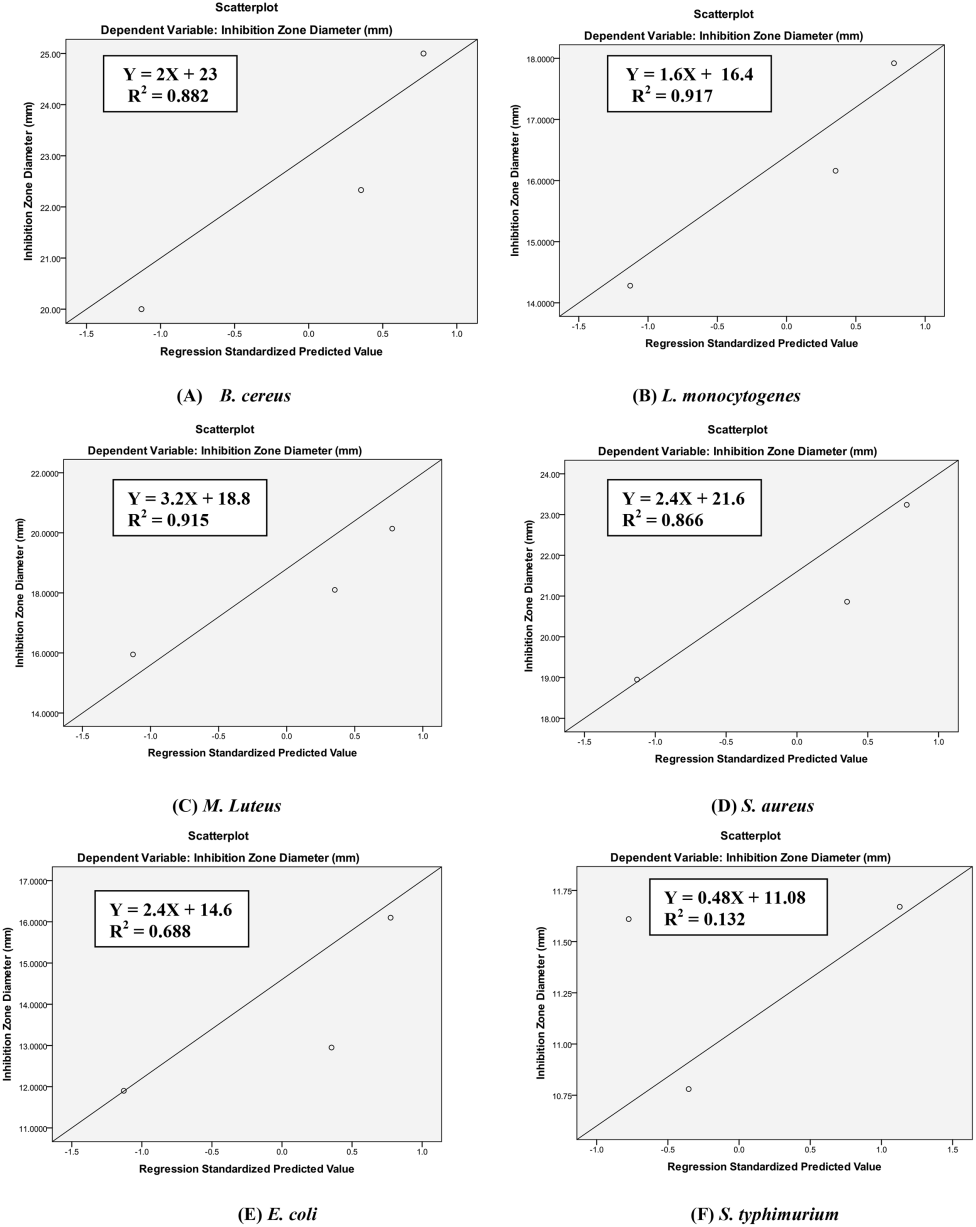
Relationship between total phenolic content and antibacterial activity of activeessential oils (coriander, cumin and mustard seed oils) against food-borne bacteria.

**Fig 3:**
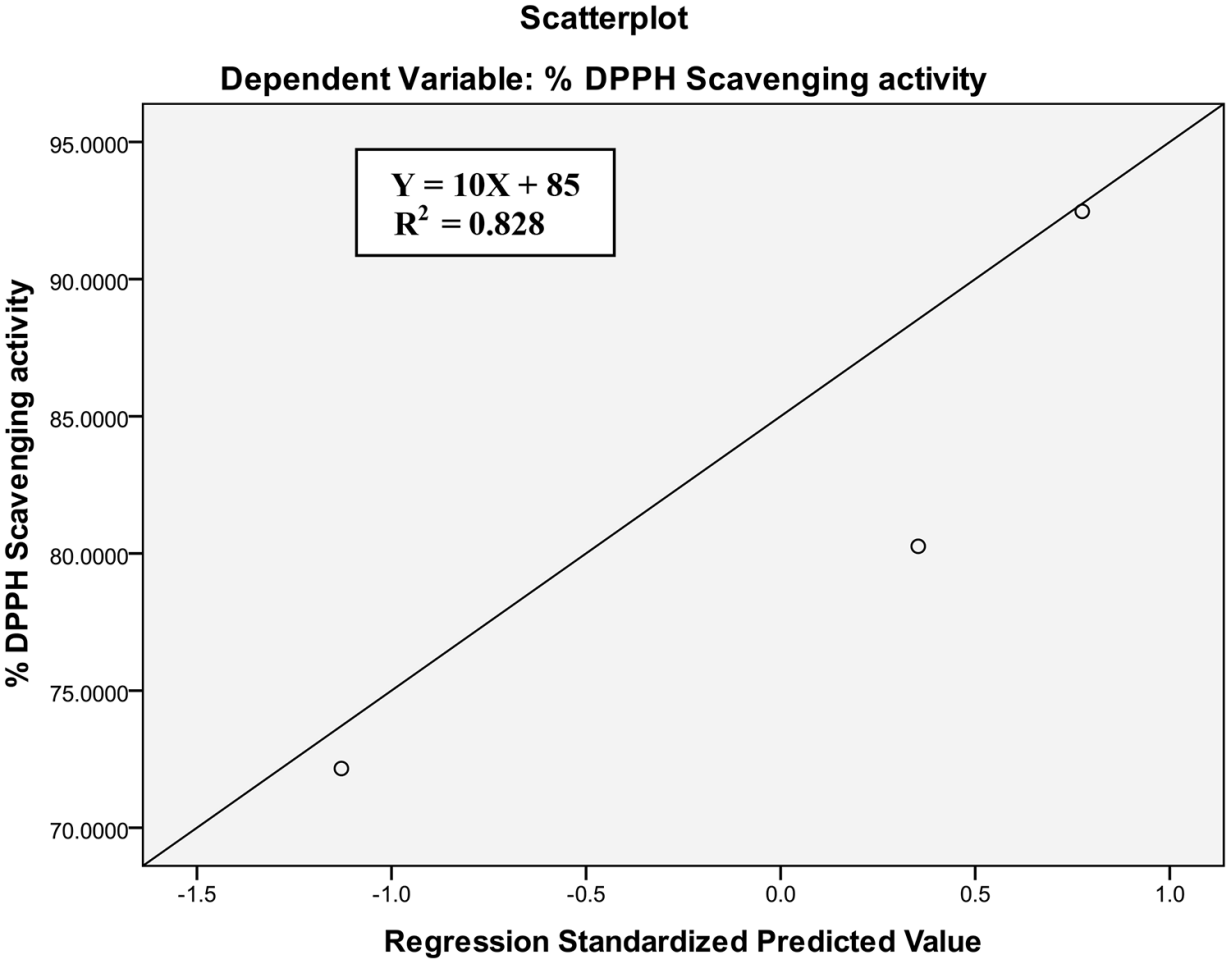
Relationship between total phenolic content and DPPH free radical scavenging activity of active essential oil.

Biological activities related to antibacterial and antioxidant activities may be correlated with total phenolic content [26, 27]. In the present study, an attempt has therefore been made to find out possible correlation between total phenolic content and antibacterial as well as antioxidant activity of active essential oils. A high positive correlation was observed between total phenolic content of essential oils and their antioxidant activity (R^2^ = 0.828) as well as antibacterial activity against most of the studied bacteria (R^2^ = 0.688–0.917) except *S*. *typhimurium* (R^2^ = 0.132) (Figs 2 & 3) which implied that phenolic compounds may have significant role on the antibacterial activity against most of the studied bacteria as well as antioxidant activity. The low R^2^ value (0.132) in case of *S*. *typhimurium* indicates that non-phenolic compounds may be effective against this bacteria.

Bioautography is an assay of target-directed isolation of active molecules on chromatogram. It combines thin layer chromatography (TLC) with bioassay *in situ*, facilitating the localization and target-directed isolation of active constituents in complex matrices of plant extract. This assay has been considered as the most efficacious assay for the detection and isolation of antimicrobial and antioxidant compounds. Thus TLC-bioautography offers the simplest mean of bioassay-guided lead discovery from natural products [19].

In our study, TLC-bioautography of coriander seed oil showed the presence of four antibacterial (R_f_: 0.20, 0.35, 0.61, 0.80) and three antioxidant (R_f_: 0.35, 0.58, 0.80) constituents. On the other hand cumin seed oil contains three antibacterial (R_f_: 0.40, 0.53, 0.70) and two antioxidant (R_f_: 0.53, 0.77) constituents. Intriguingly two constituents in coriander seed oil corresponding to R_f_ values 0.35 and 0.80 as well as one constituent in cumin seed oil corresponding to R_f_ value 0.53 exhibit both antibacterial and antioxidant activities (Table 6).

**Table 6:** Retention factor (R_f_) values of active antibacterial and antioxidant constituents isolated from coriander and cumin seed oils by TLC-bioautography.

Antibacterial compounds Essential oils		Antioxidant compounds Essential oils	
Coriander(R_f_)	Cumin(R_f_)	Coriander(R_f_)	Cumin(R_f_)
0.20	0.40	0.35	0.53
0.35	0.53	0.58	0.77
0.61	0.70	0.80	-
0.80	-	-	-

A combination study with these three isolated constituents (two from coriander seed oil (R_f_: 0.35, 0.80) and one from cumin seed oil (R_f_: 0.53) on both antibacterial and antioxidant potential was performed. It was observed that out of two possible tested combinations only one combination between isolated compounds of coriander seed oil (R_f_: 0.35) and cumin seed oil (R_f_: 0.53) exhibited both synergistic antibacterial and antioxidant activities (Table 7).

**Table 7:** Antibacterial and antioxidant combination effects of isolated bioactive components*.

**Food-borne bacteria**	**Antibacterial combinations**					
	**Coriander (R**_**f**_ **: 0.35)(A) + Cumin (R**_**f**_ **: 0.53) (B)**			**Cumin (R**_**f**_ **: 0.53) (B) +Coriander (R**_**f**_ **: 0.80) (C)**		
	**FIC**	**FICI**	**Remarks**	**FIC**	**FICI**	**Remarks**
***B*. *cereus***	0.031 (A)	0.093	S	0.031 (B)	0.781	ADD
	0.062 (B)			0.750 (C)		
***L*. *monocytogenes***	0.093 (A)	0.155	S	0.093 (B)	0.718	ADD
	0.062(B)			0.625 (C)		
***S*. *aureus***	0.125 (A)	0.187	S	0.125 (B)	0.625	ADD
	0.062 (B)			0.500 (C)		
***E*. *coli***	0.125 (A)	0.250	S	0.125 (B)	0.850	ADD
	0.125 (B)			0.725 (C)		
**Antioxidant combinations**						
**Treatment**		**IC**_**50**_ **(μg/ml)**	**CI**_**1**_ **= (D)**_**1**_ **/ (Dx)**_**1**_	**CI**_**2**_ **= (D)**_**2**_ **/ (Dx)**_**2**_	**CI = CI**_**1**_ **+ CI**_**2**_	**Remarks**
**Coriander (R**_**f**_ **: 0.35)+ Cumin (R**_**f**_ **: 0.53)**		9.95	0.24	0.33	0.57	Synergistic
**Coriander (R**_**f**_ **: 0.80)+ Cumin (R**_**f**_ **: 0.53)**		19.30	0.50	0.50	1.00	Additive

*R_f_: 0.35, 0.80 and 0.53; S: Synergistic, ADD: Additive

The concept of DART-MS analysis is different from GC-MS analysis. In the majority of cases, the time required for analysis of one sample using GC-MS is about 15–30 minutes, therefore changes in the analytical conditions may cause a drift in the results [28, 29] whereas due to the rapidness of DART-MS (only a few seconds per analysis of one sample) any changes of the analytical conditions are minimized from one sample to another. Hence the problems occur during GC-MS analysis can successfully be avoided by DART-MS analysis. In addition, DART-MS spectral fingerprints could be used for differentiation without any statistical evaluation [30]. Besides, DART-MS analysis provides the characteristics m/z values of active constituents which can be further transferred into their exact molecular weights and suggested elemental formulae of them for identifying the lead compounds [31].

For identification of bioactive compounds, the active constituents corresponding to R_f_ values 0.35 and 0.53 that showed synergistic antibacterial and antioxidant activities in combination, were subjected to DART-MS analysis. DART-MS profiling of bioactive compounds revealed that the compound isolated from coriander seed oil corresponding to R_f_ value 0.35 was linalool and the compound from cumin seed oil corresponding to R_f_ value 0.53 was p-coumaric acid (Table 8). The identified compounds were then subjected to HPLC analysis for quantification. Quantitave HPLC analysis revealed that coriander seed oil contains 68.69% linalool and cumin seed oil contains 7.14% p-coumaric acid (Table 8).

**Table 8:** Qualitative and quantitative analyses data of bioactive compounds* from coriander and cumin seed oil using DART-MS and HPLC.

DART-MS					HPLC	
R_f_ value**	Measured mass	Calculated mass	Molecular formula	Identified compound	RT	% content
0.35	154.19025	154.24932	C_10_H_18_O	Linalool	8.50	68.69
0.53	164.04930	164.0701	C_9_H_8_O_3_	*p*-Coumaric acid	12.40	7.14

* R_f_: 0.35, 0.53;

**Toluene: Ethyl acetate (95:5 v/v);

RT: Retention time

The brine shrimp lethality assay is considered a useful tool for preliminary assessment of toxicity. This assay has advantages of being rapid (24h), inexpensive and simple. It easily utilizes a large number of organisms for statistical validation. Artemia nauplii have been suggested for use as a model for several preliminary evaluation of pharmacological and ecotoxicological activities of compounds or extracts of greater complexity. According to Meyer et al [23] crude plant extract is toxic (active) if it has an LC_50_ value of less than 1000 μg/ml while non-toxic (inactive) if the LC_50_ value is greater than 1000 μg/ml. In the present study coriander/cumin seed oil combination was subjected to brine shrimp lethality assay to evaluate their cytotoxic potential, if any. It was observed that coriander/cumin seed oil combination failed to show any toxic or mortality effect at recommended dosage level with 24h LC_50_ value of 4945.30 μg/ml (Table 9) which indicates that the coriander/cumin seed oil combination is non-toxic and can be used in food ordinarily.

**Table 9:** Cytotoxic potential of coriander / cumin seed oil combination using brine shrimp lethality assay.

Dose (μg/mL)	Coriander/cumin seed oil combination			
	No. of nauplii taken	No. of nauplii alive	% Mortality	LC_50_ (μg/ml)
Control	10	10	0	4945.30
100	10	10	0	
200	10	10	0	
400	10	10	0	
800	10	9	10	
1600	10	8	20	
3200	10	6	40	
6400	10	4	60	

Furthermore, MTT assay is a rapid and high accuracy colorimetric approach that widely used to determine cell growth and cytotoxicity, particularly in the development of new drug. It measures cell membrane integrity by determining mitochondrial activity through enzymatic reaction on the reduction of yellow tetrazolium MTT to a purple formazan. So the amount of formazan produced reflected the number of metabolically active viable cells [32]. The cytotoxic effect of coriander/cumin seed oil combination was investigated *in vitro* on human normal colon cell line using MTT assay. The results of MTT assay showed that coriander/cumin seed oil combination failed to show any cytotoxic effect on human normal colon cell line at recommended dosage level and the IC_50_ value was found to be > 1000 μg/ml (Table 10) which indicates that coriander/cumin seed oil combination is safe against human intestinal cells.

**Table 10:** Cytotoxic potential of coriander/cumin seed oil combination using MTT assay.

Treatment	Concentration (μg/ml)	% cell death (72 h)	IC_50_ (μg/ml)
Coriander/cumin seed oil combination (1:1)	0	0	> 1000
	15.6	0	
	31.2	0	
	62.5	8.80 ± 1.16	
	125	10.90 ± 0.77	
	250	22.05 ± 2.86	
	500	27.86 ± 3.15	
	1000	33.17 ± 1.87	

Values are mean ± SD of triplicate experiments

## Conclusion

Thus coriander/cumin seed oil combination exhibited both synergistic antibacterial and antioxidant activity and may be used as a potential source of safe and potent natural antibacterial and antioxidant agents in pharmaceutical and food industries. Their synergistic interactions may increase their antibacterial and antioxidant efficacy at sufficiently low concentration which may reduce their adverse side effects and facilitate their use in food preservation system. Chemical analysis revealed that linalool from coriander seed oil and *p*-coumaric acid from cumin seed oil were the bioactive compounds responsible for both synergistic antibacterial and antioxidant activities. Further studies on their application in food ingredients and mechanism of action are needed to strengthen their practical applications in food system. This work may give the researchers a potential introduction to future research on these aspects. To the best of our knowledge, this is the first report on synergistic antibacterial and antioxidant activities of essential oils of spices and herbs in combination.
